# Elbow Matching Accuracy in Young and Elderly Humans under Unusual Mechanical Constraints

**DOI:** 10.3389/fnins.2016.00520

**Published:** 2016-11-16

**Authors:** Vera L. Talis, Yuri S. Levik

**Affiliations:** Institute for Information Transmission Problems (Kharkevich Institute), Russian Academy of ScienceMoscow, Russia

**Keywords:** elderly, position sense, elbow matching, elbow joint support

## Abstract

Experiment was carried out to study the proprioception accuracy of elderly (61–83 years old) and young (22–36 years old) subjects during contralateral elbow matching in sagittal plane. The subjects performed the task under ordinary condition and under experimental condition (matching forearm attached to the rocking cylindrical platform of low (LS), or high (HS) height, so that the elbow flexion was associated with tilting movement of the support and with backward movement of the upper arm). Control matching of young and elderly subjects does not differ significantly in terms of constant and absolute error. First block of LS and HS induced absolute error increase and matching arm velocity decrease in both groups, but in the second block of matching on rocking supports both arms velocity of elderly subject decreased and absolute error of elderly subjects toward the second block of rocking condition appeared lower than those of young subjects. Aftereffect of the restricted matching could be observed in elderly as a significant increase of matching arm velocity and corresponding constant error increase. It could be concluded that under unusual mechanical constraints elderly subjects tended to use “conservative” strategy followed by significant aftereffect toward the final ordinary support condition.

## Introduction

With increasing age many proprioceptively guided movements of upper limb deteriorate, for example tapping, aiming, and tracing movement slow down and become less accurate (Stelmach et al., 1988; Lovelace and Aikens, 1990). In spite of this age-related decline in motor performance explained by the overall slowing in cognitive, motor, neural and perceptual processes (Salthouse et al., 1991; Desrosiers et al., 1996; Salthouse, 1996; Erim et al., 1999; Fujiyama et al., 2013), the preserved ability to learn new skills for elderly people in visual-motor transformations task is shown (Chaput and Proteau, 1996; Etnier and Landers, 1998; Bock and Schneider, 2001, 2002; Roller et al., 2002).

The common methods to measure position sense is the matching experiment, where the accuracy of a target joint angle reproduction is analyzed in the absence of vision (Worringham and Stelmach, 1985; Gooey et al., 2000). It was shown that limb matching accuracy deteriorated substantially in the elderly (Stelmach and Sirica, 1986; Goble et al., 2009, 2012; Goble and Brown, 2010). In order to evaluate subjects' capacity of adaptation to unusual mechanical constraints we have developed the experimental design where the matching elbow was fixed on a rocking platform of different heights (Talis and Levik, 2011). In that paper we have shown that position sense of young healthy subjects is capable of quickly adapting to unusual angle-torque relationship.

The objective of the present study was to determine whether the influence of unusual elbow support conditions on the elbow angle matching is similar in young and elderly subjects, and to investigate the specificity of the adaptation process to the new mechanical constraint in young and elderly group across two experimental blocks. It is assumed (Worringham and Stelmach, 1985) that in ordinary condition the subjects elaborate the sensation of elbow joint angle mainly based on forearm weight, but on rocking supports the sense of movement is different from “static” position sense (Cordo et al., 2000). We hypothesize, that elderly subject would exhibit higher variable and constant errors than young during initial matching in ordinary condition. We also predicted that elderly and young subjects being restricted in unusual mechanical constrains would show comparable adaptability to new matching condition. The study if the ability of elderly subject to adapt to the unusual mechanical constrains and to the modified sensory inputs could be useful because elderly subjects are the target population for neuroreabilitation.

## Materials and methods

### Subjects

Twelve healthy elderly subjects (five males and seven females, mean age = 72.2 ± 4.96, range 61–83 years) and twelve healthy young controls (seven males and five females, mean age = 30.5 ± 5.50, range 22–37 years) participated in the study. All subjects were right-handed. None of the subjects reported on sensorimotor or other neurological deficits and had normal or for corrected-to-normal vision. All elderly subjects lived independently in the community. Subjects provided written, informed consent before participating in the study and the institutional review board at Institute for Information Transmission Problems approved all experimental procedures.

### Apparatus

**Figure 1:**
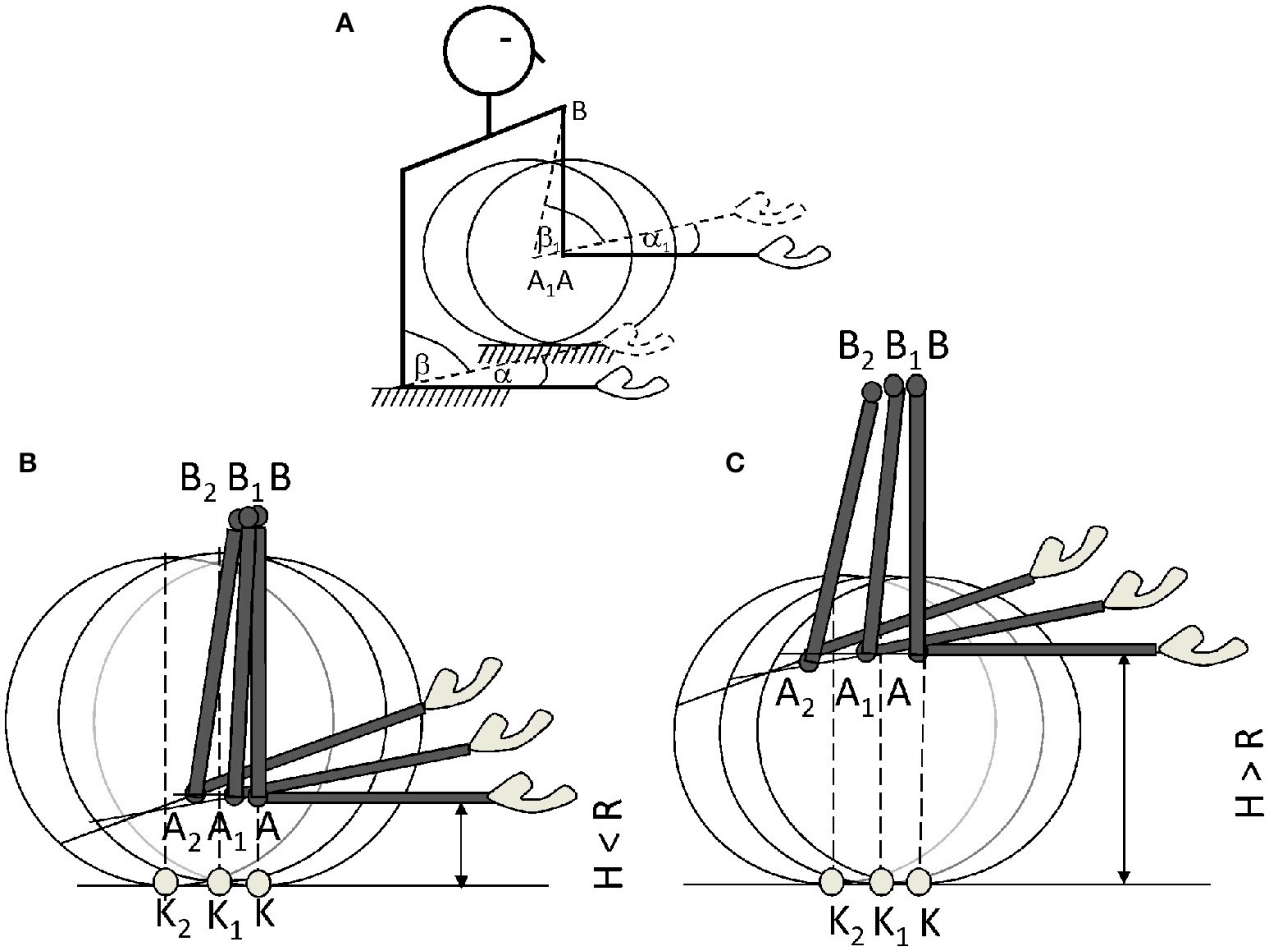
**A Schematic diagram of the experimental situation: α–reference forearm inclination angle, β–the corresponding elbow joint angle of reference arm, α_1_–the matching forearm inclination angle, β_1_–the corresponding elbow joint angle of matching arm**. Elbow joint position during elbow flexion on the low **(B)** and high **(C)** rocking supports. Note, that initially the elbow joint center **(A)** projects on the line of contact between the desk and the rocking support (K), During elbow flexion the rocking platform rotates and elbow joint center moves backward from A to A_1_ and then to A_2_, the center of shoulder joint moves from B to B_1_ and then to B_2_. Correspondingly, the point of contact between the rocking support and the desk translates backward from K to K_1_ and then to K_2_. The projection of the point A_1_ on the ground falls in front of K_1_ on the high rocking support (HS) and behind of K_1_ on the low rocking support (LS) during elbow flexion: this corresponds to a change in the momentum arm of the reaction force.

The procedure was described before (Talis and Levik, 2011) and it is briefly presented here. Subjects sat in a chair with the trunk restrained. Each forearm was placed on a separate desk, with the height of the chair being adjusted for individual upper arm length to avoid shoulder elevation. Right forearm was always placed on a rigid support; the left forearm was either on a rigid support, or on a rocking platform at the same height. The bottom of the rocking platform had a form of a cylindrical segment (radius of cylindrical segment 19 cm) so that the elbow flexion was accompanied by platform rolling (Figure 1).

To manipulate the angle-torque relationship during the elbow flexion, rocking supports of two different heights were used. The high rocking support (HS) had a height of 30 cm and the low rocking support (LS) had a height of 15 cm. Thus, on the contrast to LS, on the HS the torque generated by the reaction force relative to the elbow joint counteracted the torque generated by the forearm weight, thus decreasing a loading of the elbow flexors.

### Procedure

Subjects were instructed to flex the right (reference) elbow slowly at the “Go” signal, stop movement at the “Stop” signal and maintain the final position. The instruction was to reproduce the elbow angle of the right (reference) arm as he/she perceived it by his/her left (matching) arm. The instruction was the same for all experimental conditions. Matching accuracy requirements were stressed.

Prior to the experiment five practice trials were given to the subjects. During these trials both arms were placed on the rigid support. While practicing the experimenter monitored 3D elbow angles of the subject's both arms in order to provide the subject by the feedback if the reference arm velocity was out of 10–15°/s range.

The subjects were naive about the experimental protocol. Before the trials on the rocking support, two practice trials on the rocking support were performed.

Subjects completed 2 blocks of trials under ordinary or rigid support condition (arms-in-front position with the hands aligned and both forearms moved from the table, RS) and unstable support conditions (HS, and LS) with reference angles (70–75° and 65–70°) randomly distributed within each block of trials. Each experimental block comprised 9 trials, with the inter-trial interval of 10 s. The order of the two blocks of LS and HS conditions were counterbalanced between subjects. RS blocks were always the first (RS1) and the last (RS2) in the experiment.

### Data acquisition and analysis

The kinematics of both arms was recorded by an Optotrak system at a sampling rate of 100 Hz. Three infrared-emitting markers were placed on (1) shoulder (tip of acromion process), (2) elbow (lateral epicondyle) and (3) wrist (styloid process of the radius) of each arm. The marker position time-series were smoothed offline using a 4th-order Butterworth filter with a low-pass cutoff frequency of 10 Hz. The filtered time series was used to calculate the 3-D elbow angle and its first derivative (angular velocity) using the MATLAB computing environment.

Three parameters of matching accuracy were computed–constant error (CE), variable error (VE), and absolute error (AE). The CE was calculated as the difference between the reference and the matching elbow angle at the end of the movement. Positive and negative CE values indicated that the matching elbow was over-flexed (overestimation) and under-flexed (underestimation), respectively. AE was calculated as an absolute value of CE. VE was calculated as constant error dispersion in a course of session. Additionally, peak velocity of reference and matching elbow flexion were estimated for each subject in both groups.

### Statistical analysis

To evaluate the effects of condition on CE, VE, and AE the one-way ANOVA with the factor “condition” (RS1, HS1, LS1, RS2, HS2, LS2, RS2) was used in each group. To evaluate the effects of condition and age on CE and velocity the two-way ANOVA with the first factor “condition” and the second factor “group” (young, elderly) was used. When significant effects had been found, *post-hoc* Tukey's testing was conducted to identify the loci of these effects. The level of statistical significance was set at 0.05.

## Results

### Constant error

**Figure 2:**
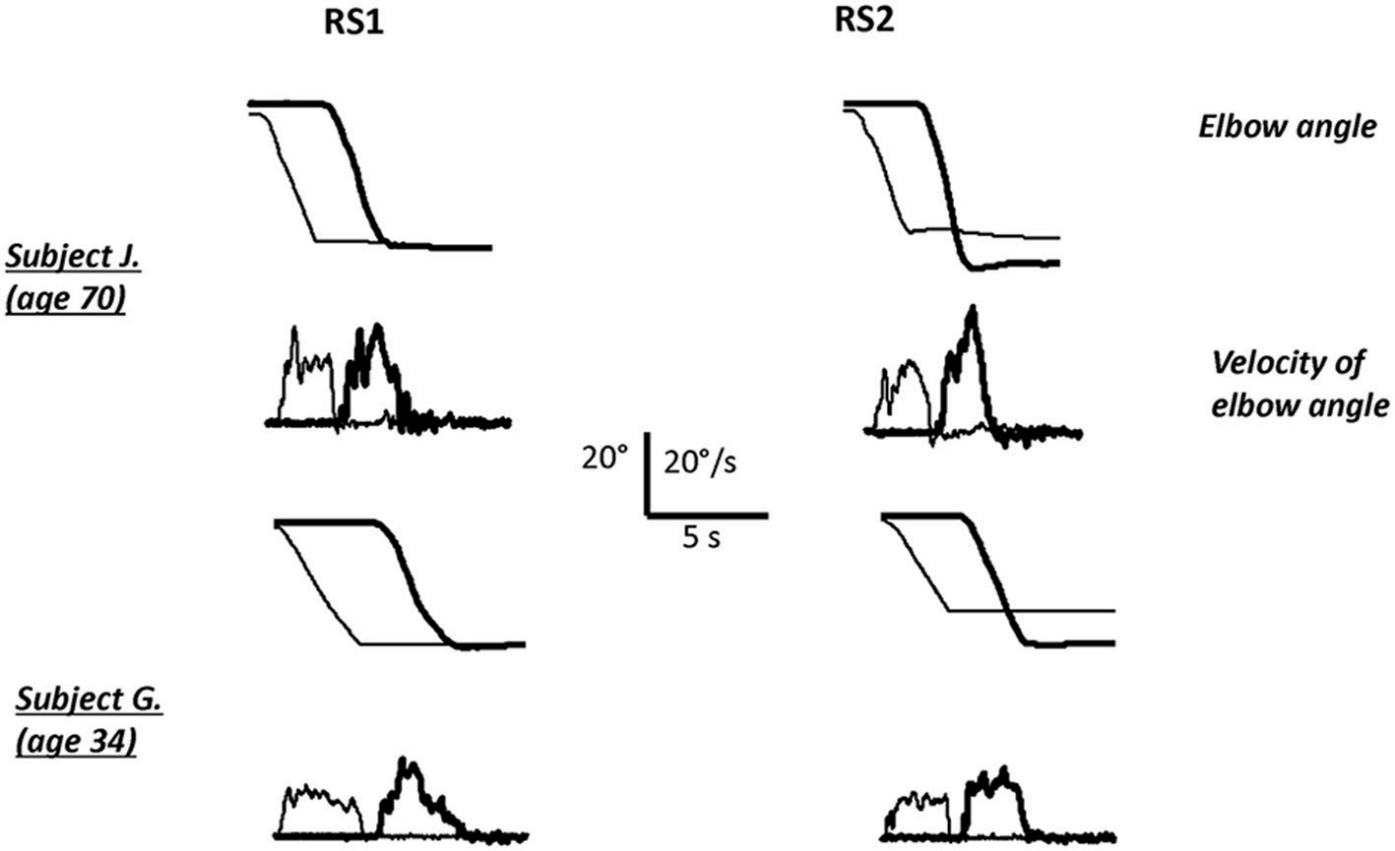
**Elbow angle and velocity on rigid support in the first block (RS1) and in the second block (RS2) for one young and one elderly subject**. The thin line–reference arm, thick line–matching arm.

**Figure 3:**
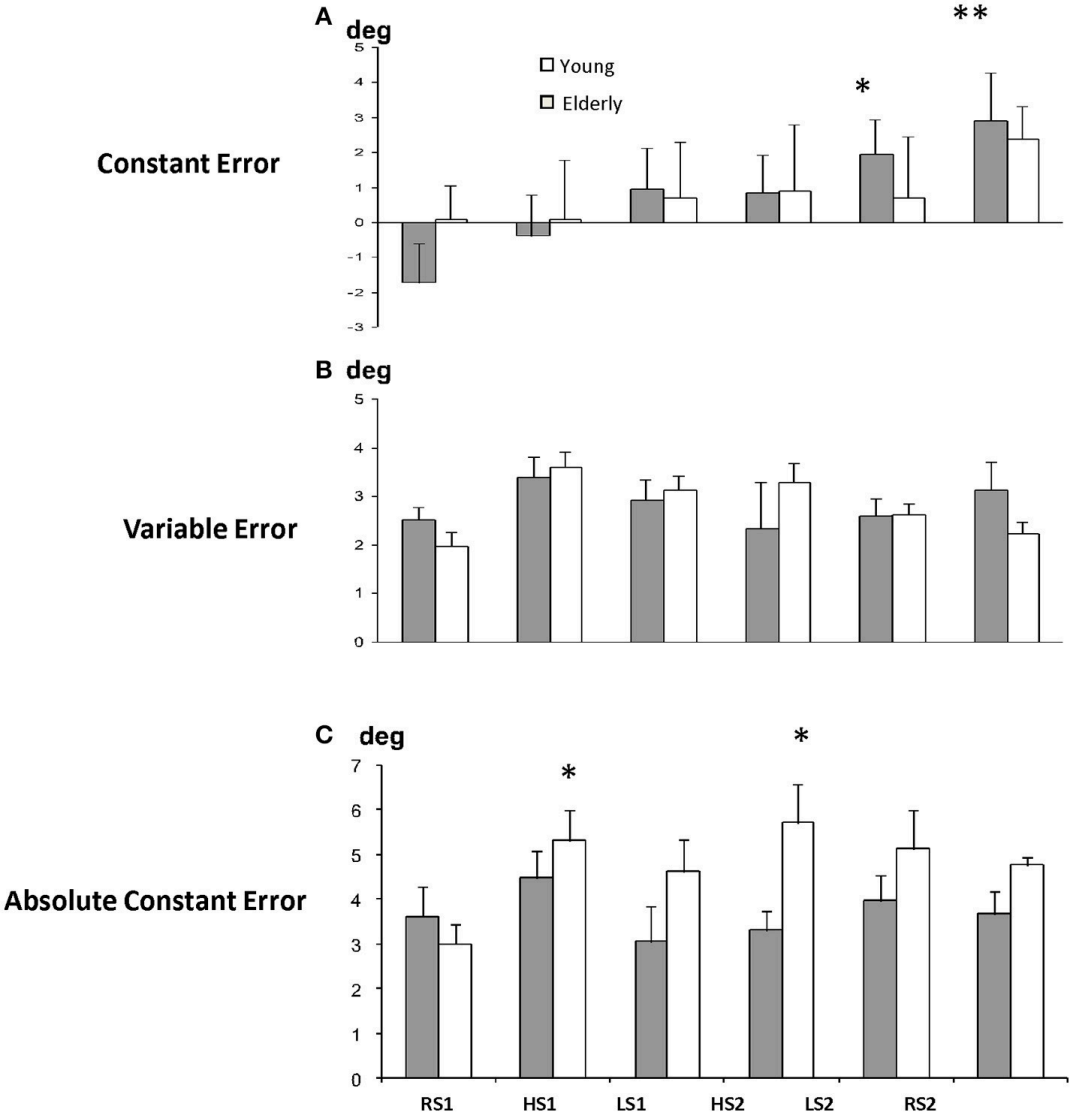
**Average group data of constant, variable and absolute errors (mean ± SE) in elderly and young subjects**. RS1, (rigid support); HS1, (high support); LS1, (low support). conditions in the first block and RS2, HS2, LS2–in the second block of the experiment. *p < 0.05, **p < 0.01.

The data acquired from one representative young and one elderly subject during initial and final performance in RS conditions are shown in Figure 2, the averaged data are represented in Figure 3. Initial performance in RS1 shows a tendency of elderly subjects to undershoot the reference elbow angle (−1.71 ± 1.09° in elderly in comparison with 0.07 ± 0.97° in young group), but this difference was not significant, also elderly subjects were slightly more variable in their initial performance (2.46 ± 0.26° in elderly group in comparison with 1.97 ± 0.27° in young group in RS1 condition).

The results of two-way ANOVA revealed no significant difference of CE between groups (*P* = 0.99), also the main effect of condition was significant [*F*_(5, 55)_ = 3.51, *p* = 0.008]. Follow-up one way ANOVAs for CE verified a significant effect of condition in elderly group. It shows that elderly subjects began to overshoot significantly the reference elbow angle toward the second block of trials [ANOVA, *F*_(5, 55)_ = 4.3, *p* < 0.005]. *Post-hoc* test revealed that CE in LS2 and RS2 was significantly higher than in RS1 condition in elderly group (*p* < 0.05 and *p* < 0.01, Figure 2A).

### Absolute and variable errors

The average group data of CE, VE, and AE in both groups of subjects shows the tendency in elderly group to undershoot the elbow angle and to be more variable in their initial performance on RS. Figure 3C shows that the first block of elbow matching on the high rocking support (HS1) produced similar disturbances in both age groups–AE increased and approached the level of significance in young group [*F*_(5, 55)_ = 3.19, *p* < 0.05]. The second block of matching on the high rocking support induced further deterioration of matching accuracy in young group—the AE increased from 3.0 ± 0.46° in RS1 condition up to 5.7 ± 0.88° in HS2 condition (*post-hoc, p* < 0.01), but not in elderly group (from 3.6 ± 0.68° in RS1 to 3.3 ± 0.44° in HS2). The results of two-way ANOVA revealed that the main effect of age was significant [*F*_(1, 41)_ = 8.27, *p* = 0.015]. Thus, elderly subjects produced the matching toward the second block of rocking condition with lower absolute error, than young subjects.

### Velocity of reference and matching arm

**Figure 4:**
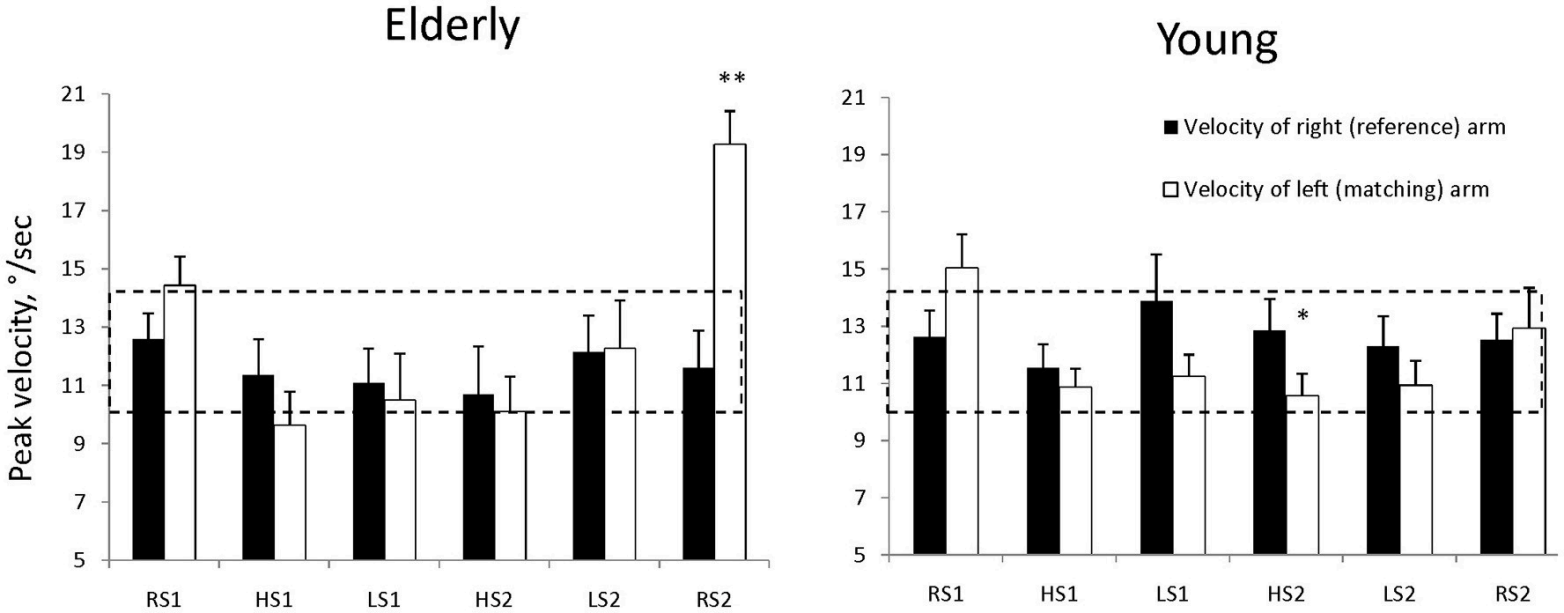
**Average group data of peak velocity (mean ± SE) of matching and reference arm motion in young and elderly group of subjects**. *p < 0.05, **p < 0.01. The dashed box shows the limits of velocity of the elbow movement that the subjects were instructed to keep. *p < 0.05, **p < 0.01—in comparison with the initial performance on rigid support (RS1).

The average group data of arms' velocity are shown in Figure 4. It can be seen, that the velocity of reference (right) arm movement was inside the instructed range (approximately 11–13°/s) across all conditions in both groups. The mean average velocity of matching (left) arm movement was 14.4 ±1.0°/s in elderly and 15.0±1.2°/s in young subjects.

The results of two-way ANOVA revealed that there was no main effect of age on velocity of matching arm. The main effect of condition on matching arm velocity was significant [*F*_(5, 45)_ = 18.66, *p* < 0.001] and age-condition interaction was also significant [*F*_(5, 40)_ = 3.42, *p* = 0.01]. In elderly group *post-hoc* reveal the increase of matching arm velocity in the RS2 condition (*p* < 0.01) and in young group decrease of matching arm velocity in HS2 condition. The significant increase of matching arm velocity from 14.4 in RS1 to 19.3°/s in RS2 condition is in contrast with the young subjects' data, whose matching arm velocity was about the same in the first and the last RS condition (15.0 and 13.0°/s).

During initial performance in ordinary condition (RS1) 7 out of 12 young and 6 out of 12 elderly subjects had the correlation coefficient between right and left elbow velocity more then 0.5 and this coefficient has decreased in these subjects in the last ordinary condition performance (RS2) (from 0.7 ± 0.07 to 0.3 ± 0.12, *t*-test < 0.01 in young and from 0.7 ± 0.04 to 0.2 ± 0.16, *t*-test < 0.05 in elderly). The correlation coefficient between velocity of matching arm and CE in elderly subjects was similar in RS1 and RS2 conditions (*r*^2^ = 0.24 ± 0.07 in RS1 and 0.24 ± 0.19 in RS2) in contrast to young subjects whose correlation between velocity of matching arm and CE was low in the RS1 (*r*^2^ = 0.14 ± 0.3) and became significantly higher in the RS2 (*r*^2^ = 0.35 ± 0.3, *p* < 0.05 of z-score between absolute values of the corresponding correlation coefficients).

## Discussion

In this paper we addressed the elbow position sense in elderly subjects. We explored the contralateral elbow matching paradigm, where the matching elbow was fixed on a rocking platform of two different heights so that the elbow flexion was associated with tilting movement of the support and the angle/torque relationship changed depending on the height of the platform. We analyzed CE and AE of contralateral elbow matching under unusual mechanical constraint. Our data showed no significant difference in accuracy of initial performance on rigid support between young and elderly subjects. In the first blocks of unstable conditions the CE and AE in both groups were also comparable; the second blocks on high rocking supports induced variability increase in young, but not in elderly group and the difference between groups became significant. Elderly subjects significantly overestimated the target angle during the last RS session.

### Initial performance of young and elderly subjects

In the present experiment elderly people, being not constrained on the speed, but on accuracy, made the motion with the same velocity, as younger adults. This is consistent with data of Brown (1996), who has shown that under self-selected speed conditions, movement durations in young and old groups were similar (see also Goble et al., 2012). Similar to wrist joint data by Ferrell et al. (1992) no systematic bias in judgments of elbow angle in the elderly group was found and the variability of initial performance of elderly was insignificantly higher than in the young. This data contradict the significant difference between elderly and young group in elbow matching experiment reported by Stelmach and Sirica (1986) and Adamo et al. (2007), however the mean AE of 2.9 and 3.63° for young and elderly in our study is comparable to 3.3° for young and 4.6° AE for elderly in study of Adamo et al.

### Rocking support performance of young and elderly subjects

The variability of performance progressively increased toward the second HS block in young, but not in elderly subjects. Similar practice effect in elderly people was observed by Buch et al. (2013), who studied adaptation in elderly group to gradual and sudden visuomotor distortions. This good adaptation in elderly group in spite of deteriorated proprioception could be explained by motor performance strategy different from that of young adults. For example, young subjects constantly kept the velocity of matching arm in new unstable condition lower than that of reference arm (Figure 4). On the contrary, elderly subjects having decreased the matching arm velocity in the first block on unstable support, tended to decrease the reference arm velocity and having similar velocity of reference and matching arm, made the movement less variable than young subjects (Figure 3). It is interesting, that this “conservative” strategy came out as a larger after-effect during matching in the last RS condition: velocity of matching arm in elderly subjects increased significantly (Figure 4) and the corresponding overshoot of the target angle can be observed (Figure 3A). Let us note, that the tendency to keep the matching arm velocity higher than the velocity of the reference one was immediately changed to the opposite when both the elderly and the young subjects have perceived their matching arm constrained (compare the initial performance with the first block unstable performance in Figure 4). Being for the second time in the unusual support condition young subjects have a tendency to move the matching arm even slower than the reference one. This differs from the elderly group, in which velocities of arms were comparable. Presumably the coactivation was a reason for this (Darling et al., 1989; Madhavan and Shields, 2005) and being unconstrained in RS2 block the elderly subjects overestimated the reference elbow angle more than the young subjects.

Several limitations of the present study are worth noting. Firstly, elderly subjects could be better motivated to accurate performance than the young, who might consider the matching task as very simple and did not regard rocking support constraints as essential because of more vigorous muscles. Secondly, subjects performed the bimanual task that relays on interhemispheric transfer (Talis et al., 2009) and put less demands on working memory thus decreasing the processing demands especially in elderly. Besides, elderly people in our experiment were not differentiated by working memory ability, which is shown to have effect on matching process (Goble et al., 2012). Thirdly, the proximal muscles involved in matching in our experiment are shown to be less affected by age, than the distal ones (Pickard et al., 2003; Shaffer and Harrison, 2007).

Another limitation of the present study was that younger individuals tested were less influenced by fatigue than elderly subjects thus explaining big difference between RS1 and RS2 in elderly group. Adding a control group that does RS 6 times might have revealed whether this change is due to fatigue/trials variability rather than to the constrain.

The results of the present study demonstrate that the effect of unusual mechanical constraints during matching task was less pronounced in elderly then in young subjects. Elderly demonstrate the preserved capability for taking into account the new mechanical constraints. This permits to modify the strategy of motor programming and processing of sensory information.

## Author contributions

All authors listed, have made substantial, direct and intellectual contribution to the work, and approved it for publication.

### Conflict of interest statement

The authors declare that the research was conducted in the absence of any commercial or financial relationships that could be construed as a potential conflict of interest.
